# Quantitative assessment of sensitizing potency using a dose–response adaptation of GARDskin

**DOI:** 10.1038/s41598-021-98247-7

**Published:** 2021-09-23

**Authors:** Robin Gradin, Andy Forreryd, Ulrika Mattson, Anders Jerre, Henrik Johansson

**Affiliations:** SenzaGen AB, Medicon Village, Scheelevägen 2, 22381 Lund, Sweden

**Keywords:** Assay systems, Applied immunology, Toxicology, Immunogenetics, Predictive markers, Machine learning

## Abstract

Hundreds of chemicals have been identified as skin sensitizers. These are chemicals that possess the ability to induce hypersensitivity reactions in humans, giving rise to a condition termed allergic contact dermatitis. The capacity to limit hazardous exposure to such chemicals depends upon the ability to accurately identify and characterize their skin sensitizing potency. This has traditionally been accomplished using animal models, but their widespread use offers challenges from both an ethical and a scientific perspective. Comprehensive efforts have been made by the scientific community to develop new approach methodologies (NAMs) capable of replacing in vivo assays, which have successfully yielded several methods that can identify skin sensitizers. However, there is still a lack of new approaches that can effectively measure skin sensitizing potency. We present a novel methodology for quantitative assessment of skin sensitizing potency, which is founded on the already established protocols of the GARDskin assay. This approach analyses dose–response relationships in the GARDskin assay to identify chemical-specific concentrations that are sufficient to induce a positive response in the assay. We here compare results for 22 skin sensitizers analyzed using this method with both human and LLNA potency reference data and show that the results correlate strongly and significantly with both metrics (r_LLNA_ = 0.81, p = 9.1 × 10^–5^; r_Human_ = 0.74, p = 1.5 × 10^–3^). In conclusion, the results suggest that the proposed GARDskin dose–response methodology provides a novel non-animal approach for quantitative potency assessment, which could represent an important step towards reducing the need for in vivo experiments.

## Introduction

Contact allergens are chemicals that possess the intrinsic potential to induce skin sensitization resulting in allergic contact dermatitis (ACD). The mechanisms of skin sensitization have been extensively reviewed previously^1–4^ and are summarized in an Adverse Outcome Pathway (AOP)^5^. Briefly, they include an asymptomatic induction phase involving priming of an adaptive immune response and clonal expansion of chemical specific T-cells resulting in allergic sensitization. If the sensitized subject is exposed subsequently to the same chemical, a cutaneous inflammatory reaction will be provoked that is described clinically as ACD.

Within the regulatory context, important tools for assessment of skin sensitization potential include the Guinea pig (GP) assays described in OECD TG 406^6^ [(GP Maximization Test (GPMT)^7^ and the Buehler Occluded Patch Test^8,9^], and the murine Local Lymph Node Assay (LLNA, TG 429)^10^. The LLNA is often the preferred method and provides several advantages compared with GP assays in terms of improved animal welfare, increased predictivity, and ability to provide a continuous estimate of sensitizing potency, as the chemical concentration required to elicit a threefold proliferation of T-lymphocytes (EC3 value) in the lymph node draining the site of topical application^11,12^ can be identified by interpolation from the dose–response curve. Moreover, a suite of mechanistically based new approach methodologies (NAMs), which are non-animal-based assays, have recently become available for hazard identification of skin sensitizers. Their development has been motivated by legislative mandates, such as the revision of Annex VII of the REACH regulation^13^, which makes non-animal testing the default requirement compared to animal tests within certain sectors. At the time of writing, seven such methods have been formally validated and incorporated into globally accepted test guidelines by OECD (OECD TG 442C^14^, D^15^ and E^16^).

A challenge for the complete replacement of animal studies for skin sensitization assessment, however, is that the current methods adopted by the OECD have thus far been validated only for hazard identification, and not for assessment of sensitizing potency, which, as indicated above, is a key requirement for effective risk management. Significant efforts have been made to develop in vitro and in silico methods as well as defined approaches (DA) for potency assessments, of which a majority focus on skin sensitizer GHS sub-categorization. However, as evident from a recent review article, this has proven to be a significant challenge, and the current state-of-the art strategies for such classifications exhibit predictive performances ranging between 55 and 69%^17^. Recently, a modified version of the Direct Peptide Reactivity Assay (DPRA, OECD TG 442 C^14^, the kinetic DPRA (kDPRA)^18^, has been described in a series of publications and has initially shown promising results for GHS potency sub-categorization^19–21^, but it still remains to be determined how well this approach performs on external data with an a priori defined classification threshold. Furthermore, to this end, development and regulatory implementation of NAM based strategies capable of providing continuous potency data beyond the discrete GHS subcategories also remain a topic of high priority.

GARD (OECD TGP 4.106) is an in vitro testing platform that brings novel elements to the field of regulatory toxicology by monitoring transcriptional patterns of biomarker signatures in a human dendritic-like cell line and provides machine-learning assisted classifications. The GARDskin assay monitors genes in a biomarker signature involved in immunologically associated pathways relevant to several key events (KE) in the AOP to arrive at mechanistically-based classifications^22^. The GARDpotency assay monitors a complementary biomarker signature^23^ and has been applied successfully for sub-categorization of sensitizers according to the GHS system. The assays were recently evaluated in a formal validation study, and reported cumulative predictive performances of 94% and 88%, for hazard identification^24^ and GHS potency sub-categorization^25^, respectively.

The present paper describes a novel adaption of the GARDskin protocol to support dose–response analyses to provide a quantitative and continuous measurement of skin sensitizing potency. The proposed strategy determines the minimal concentration required to exceed the binary classification threshold in GARDskin (DV ≥ 0), here referred to as the cDV_0_ concentration. Experimentally derived cDV_0_ values from the testing of a total of 29 chemicals are presented, together with their associated dose–response curves. Comparisons with relevant potency metrics reveal statistically significant correlations with both LLNA EC3 and human No Observed Effect Levels (NOEL).

## Results

### GARDskin dose–response: methodological description

**Figure 1:**
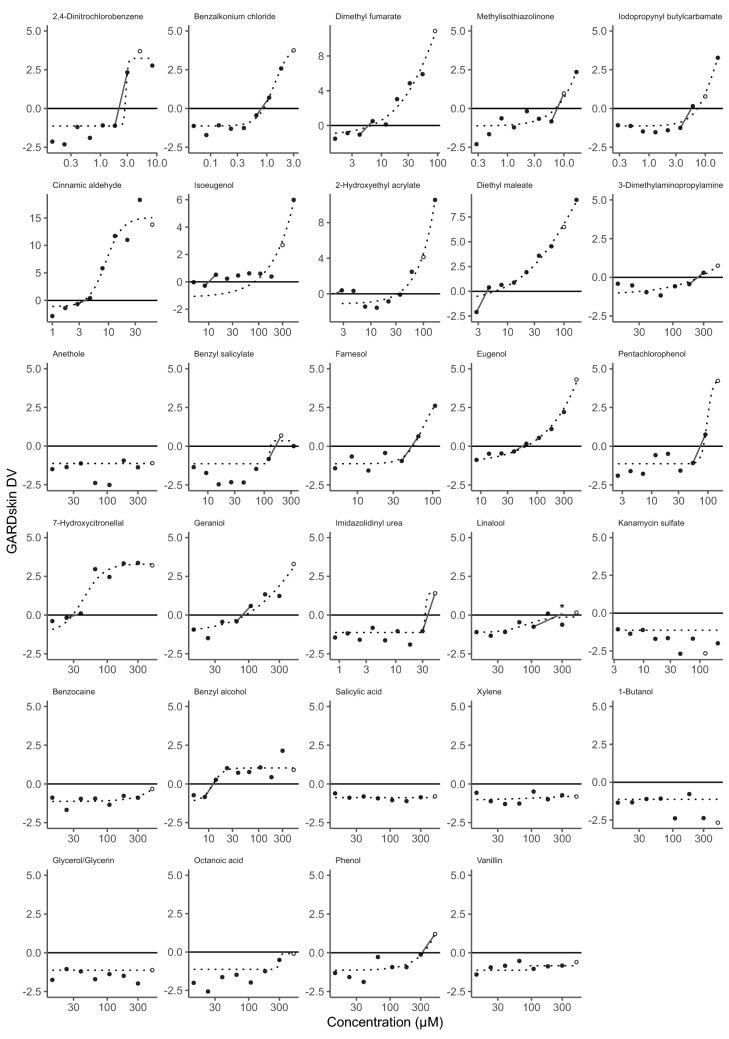
Dose–response relationships for the examined chemicals. Points represent measured response values (decision values) at respective concentrations. Points with white centers represent the GARD input concentrations. Dotted lines describe the log-logistic models, and the solid lines describe the linear interpolations. *The running median was used as positive interpolation point to reduce the potential impact of noise.

The conventional GARDskin protocol assays chemicals at single concentrations and performs hazard classifications into the categories: *skin sensitizer* and *non-sensitizer*. Although results from the assay are binary, it has been noted that the observed effects seem to be dose-dependent, demonstrating an inverse correlation between concentrations at which chemicals have been assayed, and their relative sensitizing potency. However, by itself, the GARD input concentration may not be sufficiently predictive, as observed previously^25^. Therefore, to further explore the relationship between chemical exposure concentrations and GARDskin responses, and its subsequent association with sensitizing potency, dose–response measurements were incorporated into the GARDskin protocol. Specifically, it was hypothesized that the lowest concentration required to exceed the binary classification threshold (DV ≥ 0) and generate a positive classification, referred to as the cDV_0_ concentration, would be informative of sensitizing potency. As such, the proposed dose–response measurements would also be aligned with common toxicological principles for potency assessments, where the response value (DV), the binary threshold (DV ≥ 0), and the derived cDV_0_ concentrations may be viewed as an analogue to the response value (stimulation index (SI)), the binary threshold (SI ≥ 3), and the EC3 concentrations in the LLNA assay, respectively. To evaluate this premise, a total of 29 chemicals, comprised of 7 non-sensitizers and 22 skin sensitizers of varying potency, were tested in a titrated range of concentrations. Given that the experimental setup included several novel elements, prior knowledge of the dose–response relationships’ characteristics were lacking. Therefore, the exposure concentrations were selected to evenly sample several concentrations of each chemical instead of favoring a few doses with replicates. This approach was preferred due to the reduced risk of selecting poor concentrations which could have resulted in poor estimates of the dose–response relationships^26,27^. The standard GARDskin pipeline was used to generate response values, by applying the SVM model to assign DVs to each chemical and concentration, based on the expression levels of the genes in the GARDskin biomarker signature. For each tested chemical, the assayed concentrations were plotted against the responses, i.e., the DVs, as illustrated in individual dose–response plots in Fig. 1.

### Dose–response relationships and cDV_0_ estimation

Following inspection of the acquired data, log-logistic models were fitted to each chemical to attempt to model the dose–response relationships. No-effect tests were applied to assess the presence of concentration-dependent increases in responses. The p-value for respective chemical and test is described in Table 1. As can be seen, the p-values for salicylic acid, xylene, 1-butanol, glycerol, octanoic acid, vanillin, trans-anethol, and kanamycin sulfate, agree with observations of the raw data (Fig. 1) and suggest that these chemicals did not induce increased responses in the assay over the considered concentrations. The results are further suggestive of weak responses for benzyl salicylate and benzocaine. Examination of the data showed that benzocaine did not exhibit any positive decision values, though an upward trend at the highest concentrations could potentially still be recognized, which would explain the relatively low p-value associated with the model’s fit. The two highest concentrations of benzyl salicylate generated positive responses, though with relatively low magnitudes. Remaining chemicals exhibited more distinct dose–response relationships with more significant p-values from the no-effect tests, suggesting quantifiable effects on the response values.

**Table 1:** Chemicals used to assess the GARDskin dose–response methodology and their measures of skin sensitizing potency and estimated cDV_0_ values.

No	Chemical	CAS	LLNA EC3 (%)^a^	NOEL (μg/cm^2^)^b^	GARD input c. (μM)	cDV_0_ (μM)	cDV_0_ Log-Logistic (μM) (95% CI)	cDV_0_ (mg/L)	P No-effect
1	2,4-Dinitrochlorobenzene	97-00-7	0.06	8.8	5	2.19	2.71 (2.00, 3.77)	0.443	1.05 × 10^–5^
2	Benzalkonium chloride	8001-54-5	0.1	ND	3	0.826	0.87 (0.70, 1.05)	0.350	8.15 × 10^–9^
3	Dimethyl fumarate	624-49-7	0.35	88	90	6.06	6.58 (2.88, 12.1)	0.974	1.71 × 10^–7^
4	Methylisothiazolinone	2682-20-4	0.4^28^	15	10	7.85	7.12 (2.23, 13.5)	0.904	5.32 × 10^–4^
5	Iodopropynyl butylcarbamate	55406-53-6	0.9	ND	10	5.74	7.24 (5.34, 9.78)	1.61	7.16 × 10^–7^
6	Cinnamic aldehyde	104-55-2	1.15	591	60	3.97	3.64 (2.05, 6.64)	0.524	2.21 × 10^–6^
7	Isoeugenol	97-54-1	1.35	69	300	10.4	81.8 (NA, NA)	1.70	8.62 × 10^–4^
8	2-Hydroxyethyl acrylate	818-61-1	1.56	ND	100	NA	NA	NM	1.50 × 10^–6^
9	Diethyl maleate	141-05-9	2.1^29^	1600	100	4.38	5.97 (2.20, 12.1)	0.753	2.76 × 10^–7^
10	3-Dimethylaminopropylamine	109-55-7	2.2	ND	500	251	257 (107, NA)	25.7	6.72 × 10^–3^
11	trans-Anethole	4180-23-8	2.7^30^	5510^31^	500	NS	NS	NS	1.00
12	Benzyl salicylate	118-58-1	2.85	17,717	200	164	130 (97.9, NA)	37.4	7.74 × 10^–2^
13	Farnesol	4602-84-0	4.8	2755	500	54.4	55.0 (26.4, 92.9)	12.1	1.09 × 10^–4^
14	Eugenol	97-53-0	12.9	1938	500	56.6	64.6 (57.8, 72.6)	9.29	1.96 × 10^–9^
15	Pentachlorophenol	87-86-5	20	2155	150	75.4	83.3 (52.8, 137)	20.1	7.11 × 10^–6^
16	7-Hydroxycitronellal	107-75-5	22.2	2953	500	33.1	29.0 (14.3, 43.1)	5.70	4.85 × 10^–5^
17	Geraniol	106-24-1	23.2	3875	500	82.6	88.9 (39.8, 183)	12.7	3.68 × 10^–5^
18	Imidazolidinyl urea	39236-46-9	24	2000	50	38.5	33.4 (25.6, 46.0)	14.9	3.22 × 10^–4^
19	Linalool	78-70-6	30.4	13,793	500	279	NS (71.6, NA)	43.0	9.54 × 10^–3^
20	Kanamycin sulfate	70560-51-9	NS	1874	125	NS	NS	NS	1.00
21	Benzocaine	94-09-7	NS	2000	500	NS	NS (406, NA)	NS	5.76 × 10^–2^
22	Benzyl alcohol	100-51-6	NS	5906^31^	500	12.6	12.4 (6.32, 22.8)	1.37	1.60 × 10^–3^
23	Salicylic acid	69-72-7	12.2	NS	500	NS	NS	NS	1.00
24	Xylene	1330-20-7	95.8	NS	500	NS	NS	NS	0.810
25	1-Butanol	71-36-3	NS	NS	500	NS	NS	NS	1.00
26	Glycerol	56-81-5	NS	NS	500	NS	NS (181, NA)	NS	1.00
27	Octanoic acid	124-07-2	NS	NS	500	NS	NS (224, NA)	NS	0.555
28	Phenol	108-95-2	NS	NS	500	317	328 (150, NA)	29.9	2.44 × 10^–3^
29	Vanillin	121-33-5	NS	1181	500	NS	NS	NS	1.00

*NA* Value could not be defined, *ND* Data insufficient for defining a NOEL, *NS* Non-sensitizer.

^a^Reference-data for LLNA EC3 values were, unless otherwise stated, obtained from Hoffman et al.^32^.

^b^Reference-data for human NOEL values were, unless otherwise stated, obtained from Basketter et al.^33^.

**Figure 2:**
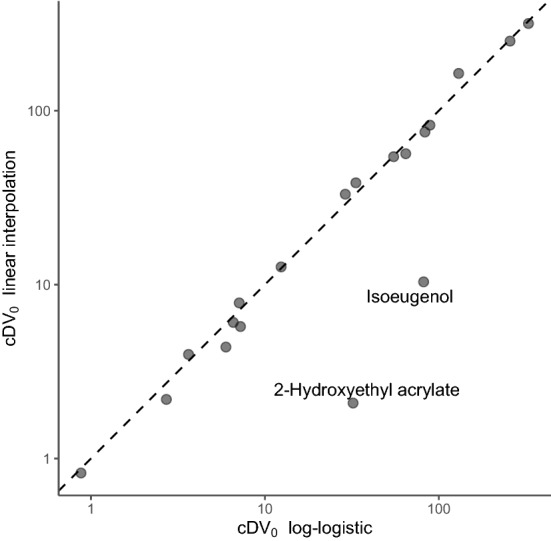
Comparison between the cDV_0_ estimation procedures. The scatter plot describes cDV_0_ values that were determined using either log-logistic modelling (x-axis) or linear interpolation (y-axis). The dashed line represents the identity line.

Next, cDV_0_ values were estimated for each chemical. Two different approaches were considered for the calculations. First, the concentrations were estimated using the already fitted log-logistic models by determining the curves’ x-axis intercepts. The log-logistic models also allowed for characterization of the uncertainty of respective cDV_0_ estimate, by calculation of approximate 95% confidence intervals, see Table 1. The log-logistic models do, however, make certain assumptions regarding the underlying data and the relationship between concentrations and response values, which might not necessarily be valid, while simultaneously requiring a relatively complex fitting procedure. Therefore, linear interpolation between two points on adjacent sides of the decision border was considered as an alternative, simpler and less constrained, method for estimation of cDV_0_. The derived concentration estimates from both approaches are compared in Fig. 2. As can be seen, both methods generally produced very similar concentrations. Three chemicals received inconsistent estimates when the two methodologies were compared. These were 2-hydroxyethyl acrylate, linalool, and isoeugenol. When examining the dose–response relationships for each of these chemicals, it can be seen that the observed discrepancy for 2-hydroxyethyl acrylate arose due to the two positive points present at the lowest exposure concentrations, giving an apparent U-shape to the overall response. Because of the unexpected appearance of these data, the chemical was excluded from further assessment. For linalool, a cDV_0_ concentration was only definable with linear interpolation. For isoeugenol, the conflicting estimates followed from the shape of the dose–response curve, which seemed to deviate from the sigmoidal shape expected by the log-logistic models. Instead, the data suggested that the chemical was able to render positive decision values at moderate magnitudes already at low concentrations, which was followed by a plateau until concentrations reached > 180 μM, at which a second increase in the decision values was observed. Given this appearance, it can be argued that the locality of the linear interpolation provides a better estimate of the true cDV_0_-value, since its fit is not constrained by a pre-determined shape of the curve. However, additional data would be useful for determining the induced dose–response curve’s shape with more certainty. Because of these observations, and the overall similarity between the results, the linear interpolation method was selected as the most appropriate technique for estimating cDV_0_, and the results described below are based on these interpolated values, which are described in Table 1.

### Comparison of cDV_0_ with established potency measures

**Figure 3:**
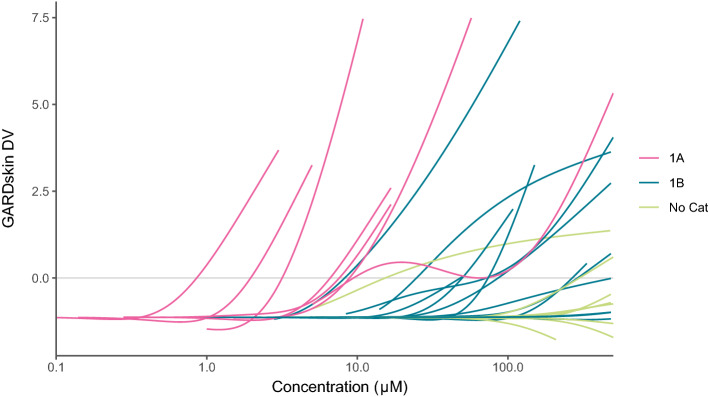
Visualization of acquired dose–response data. Each line represents a generalized additive model fitted to the dose–response data of a single chemical. The colors of the lines describe the GHS potency sub-categories, into which chemicals were assigned based on their LLNA results (1A: EC3 ≤ 2%; 1B: EC3 > 2%; No Cat: NS).

A visual representation of the experimentally derived dose–response curves for all chemicals are presented in Fig. 3, where the individual dose–response curves are colored according to their respective potency sub-category as defined by UN GHS (1A, 1B, no Cat) by the application of a 2% LLNA EC3 threshold to discriminate the stronger sensitizers from the weaker. As apparent by the figure, two of the LLNA non-sensitizers, benzyl alcohol and phenol, exhibited response-values sufficient to exceed the binary classification threshold (DV ≥ 0) at any of the assayed concentrations. The positive responses corresponded to several data points of benzyl alcohol, which is nevertheless generally considered a human skin sensitizer, and the highest evaluated concentration of phenol (500 μM). Further inspection of the figure and the generated dose–response curves clearly reveals a relationship between GHS potency and assayed concentrations, where stronger sensitizers (1A) required lower concentrations to exceed the binary classification threshold than did the weaker sensitizers (1B). Interestingly, with only a single exception for the chemical diethyl maleate, which is a CLP 1A/1B borderline chemical, a complete resolution between the GHS potency classes was observed.

**Figure 4:**
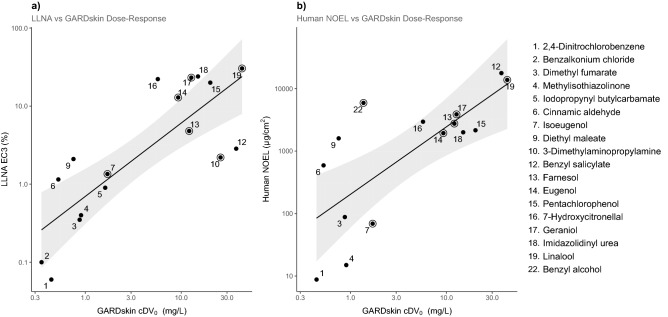
Performance assessment of GARDskin Dose–Response. Scatter plots displaying the relationship between estimated cDV_0_ values and (**a**) LLNA EC3 values and (**b**) human NOEL values. The lines represent linear regression models fitted to the data, and the shaded areas describe the 95% confidence intervals of the fits. Encircled datapoints represent indirectly acting haptens. GARDskin cDV_0_ is given in weight-based concentrations to facilitate comparison between potency measures.

To further evaluate the hypothesis that cDV_0_ values were informative of the chemicals’ relative potencies, the correlations between cDV_0_ and available potency measures were determined. First, the correlation with LLNA was considered. Figure 4 displays a scatter plot which suggests that chemicals ranked as strong sensitizers in the LLNA also seem to obtain lower cDV_0_ values. The linear association between the two metrics was quantified using Pearson’s correlation coefficient, which was determined to be 0.81 (p = 9.1 × 10^–5^; n = 17), which is indicative of a strong relationship, suggesting that GARDskin cDV_0_ was significantly associated with sensitizing potency, as defined by LLNA EC3 values.

Second, the relationship between human NOEL values and cDV_0_ values was considered and Fig. 4 shows the relationship between these two metrics. Similar to the LLNA-figure, a clear correlation can be observed. Indeed, quantification of the strength of the linear association resulted in a Pearson correlation coefficient of 0.74 (p = 1.5 × 10^–3^; n = 15), further supporting the observation that cDV_0_ values were informative of sensitizing potency.

Finally, indirectly acting haptens, i.e., chemicals that require either abiotic or biotic activation prior to being able to induce sensitization reactions, are also displayed in Fig. 4. As can be seen, the data suggest that cDV_0_ values are also informative of potency for these types of chemicals, suggesting that the GARDskin dose–response methodology is also able to assess indirectly acting haptens.

## Discussion

The ability to identify and characterize chemical hazards is a requirement for effective risk assessment and risk management. Induction of skin sensitization is a threshold phenomenon^34^, and the risk assessment procedure follow the general toxicological principles of quantitative risk assessments (QRA). The first step in this procedure is to derive a continuous prediction of skin sensitizing potency to define a concentration below which no reactions are expected to occur, a so-called No Expected Sensitization Induction Level (NESIL) value^35^, for use as a point-of-departure (POD) in the QRA. While there have been considerable efforts within the scientific community toward the development of non-animal methods capable of providing such information, animal data, preferably from the LLNA, still remain an important source of information for quantitative potency assessments. For example, it can be used in regulatory contexts to derive discrete GHS sub-categorization^11^ or in risk assessments where the NESIL value can be derived directly from the LLNA EC3 value, which correlates well with results from Human Repeated Insult Patch Testing (HRIPT)^36^. The ability to acquire such information from NAM technologies would nevertheless comprise an essential component in a non-animal toxicological toolbox for skin sensitizer assessment for the ultimate goal of replacing animal experimentation. In this context, it is worthwhile to remember that any assay developed towards a complex endpoint such as skin sensitization will likely be associated with errors, which also underscores the importance of effective post market surveillance programs for preservation of human health^37,38^.

The GARDskin assay was developed for hazard identification of skin sensitizers and has been shown to be capable of accurately discriminating skin sensitizers from non-sensitizers^24^. In this study, we extend the original GARDskin method to include dose–response analysis for the purpose of extracting information appropriate for quantitative hazard characterization. Of the 22 potential skin sensitizers included in the analysis, 19 gave rise to positive response values at some of the assayed concentrations. The three chemicals that failed to induce any detectable responses were anethole, benzocaine, and kanamycin sulphate. It is worth noting that all of these chemicals are considered to be very weak skin sensitizers, and the expected classification labels for kanamycin and benzocaine are in fact ambiguous comparing human data to LLNA. The data recorded for 2-hydroxyethyl acrylate was ambiguous which hindered the chemical’s further analysis. Additional data will be required to elucidate the dose–response curve’s actual shape and characteristics. For each of the remaining sensitizers, the lowest concentration capable of rendering a positive prediction was estimated using linear interpolation, and the cDV_0_ values were compared with potency measures available for respective chemical. It was found that cDV_0_ correlated significantly with both LLNA EC3 (*r* = 0.81, p = 9.1 × 10^–5^) and human NOEL (*r* = 0.74, p = 1.5 × 10^–3^). The magnitudes of the correlation coefficients suggested that the associations between cDV_0_ and both potency measures were strong, emphasizing the potential usefulness of the method. Moreover, the inclusion of several indirectly acting haptens among the chemicals indicated that GARDskin Dose–Response was also capable of generating meaningful cDV_0_ values for these types of chemicals. The assessment of indirectly acting haptens are important as the ability of NAMs to assess such chemicals is a point of general concern^39^.

It is also interesting to consider and compare the achieved performance figures with those obtained by other non-animal methods hitherto proposed for quantitative potency assessment. However, the number of overlapping chemicals among these assays are still minute, making such comparisons uncertain and not sufficiently informative to allow characterization of potential advantages or disadvantages of respective method. Therefore, we would like to postpone such comparisons until larger coherent sets of overlapping chemicals are available. Nevertheless, for the interested reader, performance measures for alternative methods on overlapping set of chemicals have been provided in Supplementary Data. Also, in the general context of assessing the results, it is necessary to acknowledge the difficulty in evaluating quantitative potency data due to imperfect reference data. Ideally, quantitative results from novel non-animal assays would be compared to well defined human-relevant potency references. However, such reference data are exceedingly rare and alternative measures have to be considered. In this study, we have included a comparison between cDV_0_ and human NOEL. However, NOEL values are not a proper potency measure but corresponds to a concentration where no adverse effect has been observed, meaning that it is likely that concentrations exceeding NOEL might still not induce sensitization. And while it is true that they carry information of potency, they remain an imperfect source of reference.

There are several advantages to be had when an assay provides quantitative continuous potency results compared with qualitative or discrete ones, including the ability to rank chemicals based on their relative potencies’, or the possibility to use the results as input in QRAs. This is a feature that separates the outcomes of the herein proposed GARDskin Dose–Response methodology and the previously described GARDpotency assay, which discriminates between strong and weak sensitizers based on the induced gene expression levels of a separate biomarker signature^23,25^. Thus, while the two assays might arguably provide some overlapping information, it would be expected that the GARDskin Dose–Response assay could provide relatively more information but at the cost of additional resources. Therefore, based on the information requirements of a particular test case, the two assays could still fill complementary data gaps. The generated cDV_0_ values seem to correlate strongly with both examined potency measures, suggesting that the GARDskin Dose–Response assay could constitute an early but promising non-animal method for skin sensitizing hazard characterization. Assuming that the evidence described in this paper can be further reinforced on larger chemical sets, the proposed method could act as a direct alternative to the LLNA. The experimentally derived data would also be interpreted very similarly, which should make results accessible. However, it should again be noted that the examined dataset contains a limited number of chemicals, and additional data would indeed be necessary to increase the certainty of the observed association with sensitizing potency.

Another aspect of the analysis concerns the experimental protocol and the selection of assayed concentrations. Given that this was an initial study aimed towards examining the hypothesis that the lowest concentration capable of generating a positive GARDskin prediction would carry information of skin sensitizing potency, a few unknowns regarding the experimental procedures existed. For example, the characteristics of the responses and their dependence on the chemical exposure concentration was not known. Therefore, to increase the likelihood of observing response values in the vicinity of the decision boundary, it was prioritized to evaluate multiple concentrations in contrast to a few but replicated steps. This decision can also be justified by the non-controversial assumption that some smooth function describing the relationship between concentration and response exists (which is also reinforced by the relatively large number of assayed chemicals in GARDskin at utilized concentrations that show positive responses), which could potentially be more efficiently explored by an adequate number of sampled concentrations^26,27,40^. However, given these results, the selection of exposure concentrations could likely be optimized for future experiments to reduce uncertainties in cDV_0_ estimates and to reduce time and cost expenditures. This analysis is, however, considered outside the scope of this publication since it could potentially require refinements as additional data are accumulated.

In conclusion, based on results presented in this paper, we argue that the proposed protocol, based on the validated protocol of GARDskin, provide an encouraging opportunity to derive in vitro quantitative information related to the inherent sensitizing potency of chemicals, which can be used for potency-associated rankings, for GHS potency sub-categorization as a complement to GARDpotency, or for direct incorporation into existing strategies for QRAs.

## Methods

### Chemicals

The chemicals used in this study were purchased from Sigma Aldrich (St Louis, Missouri), and their identities are listed in Table 1. They were selected to include a majority of skin sensitizers, which would allow for evaluation of the hypothesis that cDV_0_ was associated with potency. A set of non-sensitizers was also included to verify that they did not give rise to positive responses over the examined concentration ranges. The skin sensitizers were selected to form a set of chemicals with varying skin sensitizing potencies, ranging from compounds generally considered as weak sensitizers to those considered as relatively stronger.

### Cellular exposure experiments

The experimental procedures were adapted from the default protocols of the GARDskin assay. The GARDskin method has previously been described in detail and the complete protocol has been made publicly available^24^. The scientific validity and performance of GARDskin has also recently been confirmed, in a detail peer review^41^. Nevertheless, briefly described, the GARDskin assay is initiated by evaluating the cytotoxic properties of a test chemical by exposing the cell line, Senzacell (ATCC depository PTA-123875), to the chemical at a range of concentrations. The cell line is a human myeloid dendritic-like cell line, acting as a surrogate for dendritic cells. It is a stable cell line derived from MUTZ-3 (available from DSMZ, ACC 295) by adaptation to simplified growth conditions and cell maintenance protocols (WO 2019/057977), as further described in Ref.^22^. Following 24 h of chemical exposure, cells are harvested and cell viability is examined using propidium iodide staining and flow cytometry analysis. A chemical concentration capable of inducing low- to non-cytotoxic levels is then identified, where low cytotoxicity is defined as a decrease of approximately 10% in cell viability compared to unstimulated controls. This concentration is henceforth referred to as the GARD input concentration. The cytotoxicity screening ensures that chemical effects on the cells are not driven by toxicity, while also homogenizing exposure conditions for all test chemicals. Three independent exposure experiments are then performed at the GARD input concentration. Following 24 h of chemical exposure in each of the experiments, cells are harvested and RNA is isolated and quality controlled, generating three biological replicate samples. The expression levels of the genes in the GARDskin prediction signature (GPS^22,42^) are quantified using NanoString nCounter technology^43^, and the skin sensitizing hazard property of the chemical is predicted using the GARDskin prediction model. The exact identity of the genes in the GPS are available in previous publications^22^.

For the purpose of dose–response analysis in GARDskin, minor extensions to the original protocols were introduced. For this study specifically, all examined chemicals had previously been investigated in GARD-associated research and validation studies and formerly established GARD input concentrations were used^23,24,42^. Chemical-specific concentrations were selected as described in the section below. Exposure experiments in accordance with the standard GARDskin protocols were carried out at all selected concentrations. However, in contrast to the original protocols, to facilitate the acquisition and enable dose-dependent analysis, each concentration was assayed in one biological replicate. Remaining experimental steps were carried out in accordance with the default GARDskin protocol^24^.

### Concentration selection

Individual concentration steps were selected for all chemicals. Each set of concentrations was comprised of a geometric sequence with a scaling factor of 3/5. For each chemical, twelve different concentrations were initially considered during the cellular exposure experiments, where the highest concentration corresponded to two steps above the GARD input concentration or to 500 μM, which is in accordance with the standard GARDskin upper concentration limit. The general formula for calculating the different concentration levels is described in Eq. (1). Based on observed cytotoxicity following exposure experiments, eight to nine concentrations per chemical were selected for quantification and classification using the GARDskin pipeline, where concentrations inducing excessive cytotoxicity and/or the lowest evaluated concentration where excluded. The selected concentrations for all chemicals are described in Supplementary Information, and the utilized GARD input concentrations are described in Table 1.

1 $$\begin{array}{*{20}c} {c_{i} = c_{input} \times \left( \frac{3}{5} \right)^{i - 3} , \,i = \left\{ {1, 2, \ldots , 12} \right\}} \\ \end{array} ,$$

where *c*_*i*_ represent the concentration at step *i*, and *c*_*input*_ the GARD input concentration.

### Dose–response analysis

Response values were defined as the decision values generated by the standard GARDskin prediction model, a Support Vector Machine (SVM)^44^ that was trained and frozen during assay development, as described^42^.

Dose–response relationships were modelled using log-logistic relationships, as defined by Eq. (2), where parameters *c* and *d* represent the lower and the upper response limits, respectively, *b* the slope, and *e* the effective dose ED50. Additional constraints were also enforced in the fitting procedure. The lower asymptote, *c*, was kept fixed at the response value of unstimulated controls. Further, the slope parameter, *b*, was only allowed to take on negative values, thereby generating monotonically increasing curves. Models were fitted using the R-package drc^45^.

2 $$\begin{array}{*{20}c} {f\left( {x, \left( {b, c, d, e} \right)} \right) = c + \frac{d - c}{{1 + exp^{{b\left( {\log \left( x \right) - \log \left( e \right)} \right)}} }}} \\ \end{array} .$$

For each chemical, the presence of concentration-dependent response values were evaluated by comparing the fitted log-logistic model against a simpler model represented by the best fitted horizontal line (i.e., the expected shape of the data if the concentration would have no effect on the response values). The models’ relative ability to explain the data was tested using likelihood ratio tests, where a significant result indicated that the more complex model explained the data better. This test is referred to as a no-effect test in the remainder of the text.

The lowest concentration where a positive decision value would be generated, i.e., the cDV_0_ concentration, was estimated using two different approaches. In the first approach, cDV_0_ was calculated using the log-logistic models. From these models, the uncertainty of an estimate was also evaluated by approximating the values’ 95% confidence intervals using inverse regression^46–48^. The second approach for cDV_0_ estimation consisted of linear interpolation, which was performed between two points observed on adjacent sides of the decision border, see Eq. (3) below.

3 $$\begin{array}{*{20}c} {cDV_{0} = c_{ - } - \frac{{DV_{ - } *\left( {c_{ + } - c_{ - } } \right)}}{{DV_{ + } - DV_{ - } }}} \\ \end{array} .$$

where, c_−_ and c_+_ represent the concentration of the negative and the positive point respectively, and DV_−_ and DV_+_ the decision value of the negative and the positive point, respectively.

### Comparison with established potency measures

The hypothesis that cDV_0_ values were informative of skin sensitizing potency was tested by comparing the estimated cDV_0_ concentrations to already established potency measures, including LLNA EC3 values and NOEL values from human repeated insult patch tests (HRIPT). LLNA EC3 concentrations were generally collected from Ref.^32^. However, the EC3 value for methylisothiazolinone was obtained from Ref.^28^ (due to a previous incorrect reporting of LLNA-data), an EC3 value of 2.1% was used for diethyl maleate as argued in Ref.^29^, and the EC3 value of *trans*-anethol was obtained from Ref.^30^. Human NOEL values were collected from Refs.^33^ and ^31^. CLP-categories were generated from the EC3-values by applying a 2% classification threshold to discriminate between sensitizers of sub-categories 1A and 1B, as defined by the UN GHS. The strength and significance of the association between cDV_0_ and EC3 values, and cDV_0_ and human NOEL were evaluated using Pearson’s correlation coefficient on log-transformed measures.

### Visualization

Figures were created using R v4.0.2^49^ with the package ggplot2 v3.3.2^50^. To visualize the acquired dose–response data as shown in Fig. 3, generalized additive models (GAMs) were fitted to the individual chemicals’ dose–response curves using the R-package mgcv v1.8-31^51^.

## Supplementary Information

Supplementary Information.

## Data Availability

All data supporting the results are available in the manuscript or supporting materials.
