# Supplementary material for: Quantitative assessment of sensitizing potency using a dose–response adaptation of GARDskin
Source: Sci Rep. 2021 Sep 23;11:18904. doi: 10.1038/s41598-021-98247-7 (PMC8460622; doi:10.1038/s41598-021-98247-7)
Supplement: Supplementary file 1 — Supplementary Information. [file 41598_2021_98247_MOESM1_ESM.docx]

Supplementary materials

*Assayed concentrations*

**Supplementary table 1***.* Assayed concentrations and response values for the different chemicals are described.

| **Substance** | **Assayed concentration** | **Decision value** |
| --- | --- | --- |
| 1-Butanol | 500, 300, 180, 108, 64.8, 38.9, 23.3, 14 | −2.67, −2.37, −0.786, −2.39, −1.08, −1.11, −1.33, −1.35 |
| 2-Hydroxyethyl acrylate | 167, 100, 60, 36, 21.6, 13, 7.78, 4.67, 2.8 | 10.5, 4.12, 2.48, −0.0909, −0.852, −1.56, −1.44, 0.334, 0.388 |
| 2,4-Dinitrochlorobenzene | 8.3, 5, 3, 1.8, 1.1, 0.65, 0.39, 0.23, 0.14 | 2.77, 3.7, 2.33, −1.11, −1.09, −1.9, −1.19, −2.32, −2.14 |
| 3-Dimethylaminopropylamine | 500, 300, 180, 108, 64.8, 38.9, 23.3, 14 | 0.752, 0.3, −0.44, −0.576, −1.17, −0.959, −0.521, −0.41 |
| 7-Hydroxycitronellal | 500, 300, 180, 108, 64.8, 38.9, 23.3, 14 | 3.2, 3.37, 3.33, 2.46, 2.96, 0.104, −0.175, −0.39 |
| Anethole | 500, 300, 180, 108, 64.8, 38.9, 23.3, 14 | −1.11, −1.38, −0.938, −2.52, −2.39, −1.13, −1.36, −1.5 |
| Benzalkonium chloride | 3, 1.8, 1.1, 0.65, 0.39, 0.23, 0.14, 0.084, 0.05 | 3.75, 2.58, 0.7, −0.448, −1.26, −1.31, −1.09, −1.72, −1.12 |
| Benzocaine | 500, 300, 180, 108, 64.8, 38.9, 23.3, 14 | −0.323, −0.894, −0.772, −1.35, −0.945, −0.961, −1.68, −0.888 |
| Benzyl Alcohol | 500, 300, 180, 108, 64.8, 38.9, 23.3, 14, 8.4, 5.04 | 0.909, 2.14, 0.437, 1.07, 0.768, 0.717, 1.02, 0.271, −0.846, −0.727 |
| Benzyl salicylate | 334, 200, 120, 72.1, 43.2, 25.9, 15.6, 9.34, 5.6 | 0.00677, 0.687, −0.829, −1.47, −2.34, −2.32, −2.46, −1.73, −1.35 |
| Cinnamic aldehyde | 60, 36, 21.6, 13, 7.8, 4.7, 2.8, 1.7, 1 | 13.8, 18.3, 11, 11.7, 5.83, 0.421, −0.671, −1.39, −2.85 |
| Diethyl maleate | 167, 100, 60, 36, 21.6, 13, 7.78, 4.67, 2.8 | 9.22, 6.49, 4.54, 3.59, 1.93, 0.882, 0.644, 0.389, −2.09 |
| Dimethyl fumarate | 90, 54, 32, 19, 12, 7, 4.1, 2.5, 1.5 | 10.9, 5.9, 4.85, 3.02, 0.114, 0.503, −1.06, −0.878, −1.52 |
| Eugenol | 500, 300, 180, 108, 64.8, 38.9, 23.3, 14, 8.4 | 4.3, 2.21, 1.11, 0.536, 0.158, −0.341, −0.469, −0.487, −0.883 |
| Farnesol | 108, 64.8, 38.9, 23.3, 14, 8.4, 5.04 | 2.61, 0.64, −0.955, −0.44, −1.57, −0.665, −1.43 |
| Geraniol | 500, 300, 180, 108, 64.8, 38.9, 23.3, 14 | 3.29, 1.23, 1.34, 0.589, −0.412, −0.436, −1.49, −0.936 |
| Glycerol/Glycerin | 500, 300, 180, 108, 64.8, 38.9, 23.3, 14 | −1.12, −1.98, −1.49, −1.38, −1.71, −1.2, −1.05, −1.75 |
| Imidazolidinyl urea | 50, 30, 18, 10.8, 6.5, 3.9, 2.3, 1.4, 0.84 | 1.4, −1.04, −1.9, −1.04, −1.63, −0.823, −1.6, −1.18, −1.45 |
| Iodopropynyl butylcarbamate | 16.7, 10, 6, 3.6, 2.16, 1.3, 0.78, 0.47, 0.28 | 3.28, 0.777, 0.152, −1.26, −1.41, −1.53, −1.48, −1.13, −1.08 |
| Isoeugenol | 500, 300, 180, 108, 64.8, 38.9, 23.3, 14, 8.39, 5.04 | 5.98, 2.69, 0.386, 0.595, 0.619, 0.463, 0.232, 0.52, −0.284, −0.03 |
| Kanamycin sulfate | 208, 125, 75, 45, 27, 16.2, 9.7, 5.8, 3.5 | −2, −2.66, −1.69, −2.69, −1.65, −1.7, −1.11, −1.36, −1.06 |
| Linalool | 500, 300, 180, 108, 64.8, 38.9, 23.3, 14 | 0.162, −0.62, 0.0923, −0.752, −0.464, −1.09, −1.34, −1.1 |
| Methylisothiazolinone | 16.7, 10, 6, 3.6, 2.2, 1.3, 0.78, 0.47, 0.28 | 2.35, 0.969, −0.834, −0.665, −0.178, −1.22, −0.635, −1.66, −2.3 |
| Octanoic acid | 500, 300, 180, 108, 64.8, 38.9, 23.3, 14 | −0.105, −0.515, −1.25, −1.98, −1.48, −1.64, −2.57, −2.01 |
| Pentachlorophenol | 150, 90.1, 54, 32.4, 19.5, 11.7, 7, 4.2, 2.5 | 4.22, 0.75, −1.09, −1.58, −0.493, −0.579, −1.78, −1.61, −1.9 |
| Phenol | 500, 300, 180, 108, 64.8, 38.9, 23.3, 14 | 1.21, −0.114, −0.931, −0.925, −0.275, −1.88, −1.57, −1.31 |
| Salicylic acid | 500, 300, 180, 108, 64.8, 38.9, 23.3, 14 | −0.807, −0.863, −1.11, −1.05, −0.93, −0.808, −0.892, −0.61 |
| Vanillin | 500, 300, 180, 108, 64.8, 38.9, 23.3, 14 | −0.605, −0.829, −0.876, −1.04, −0.528, −0.838, −0.948, −1.4 |
| Xylene | 500, 300, 180, 108, 64.8, 38.9, 23.3, 14 | −0.816, −0.73, −0.996, −0.494, −1.27, −1.3, −1.12, −0.572 |

*Comparison of potency performance between assays*

**Supplementary Table 2**. Overlapping set of chemicals when comparing GARDskin Dose-Response’s estimated cDV_0_ values to kDPRA’s log k_max_ and hCLAT’s minimum induction threshold (MIT).

| Substance | CAS | Mw (g/mol) | LLNA EC3 (%) | LLNA pEC3 (M^-1^) | GARDskin Dose-Response cDV0 (mg/L) | hCLAT MIT (mg/L) | kDPRA log k_max_ (k_max_ in units of s^-1^M^-1^) |
| --- | --- | --- | --- | --- | --- | --- | --- |
| 2,4-Dinitrochlorobenzene | 97-00-7 | 202.55 | 0.06 | 337 | 0.443 | 1.92 | −0.56 |
| 3-Dimethylaminopropylamine | 109-55-7 | 102.18 | 2.2 | 4.64 | 25.7 | 82.1 | NA |
| 7-Hydroxycitronellal | 107-75-5 | 172.26 | 22.2 | 0.776 | 5.70 | 26.8 | −2.77 |
| Cinnamic aldehyde | 104-55-2 | 132.16 | 1.15 | 11.5 | 0.524 | 10.2 | −1.35 |
| Diethyl maleate | 141-05-9 | 172.18 | 2.1 | 8.20 | 0.754 | 64 | −1.21 |
| Dimethyl fumarate | 624-49-7 | 144.13 | 0.35 | 41.2 | 0.874 | 3.94 | −0.27 |
| Eugenol | 97-53-0 | 164.21 | 12.9 | 1.27 | 9.29 | 64.4 | −2.64 |
| Farnesol | 4602-84-0 | 222.37 | 4.8 | 4.63 | 12.1 | 21 | −3.41 |
| Geraniol | 106-24-1 | 154.25 | 23.2 | 0.665 | 12.7 | 123.03 | −3.41 |
| Imidazolidinyl urea | 39236-46-9 | 388.29 | 24 | 1.62 | 14.9 | 39.3 | −1.11 |
| Iodopropynyl butylcarbamate | 55406-53-6 | 281.09 | 0.9 | 31.2 | 1.61 | 8.15 | 0.75 |
| Linalool | 78-70-6 | 154.25 | 30.4 | 0.507 | 43.0 | 68.3 | NA |
| Methylisothiazolinone | 2682-20-4 | 115.15 | 0.4 | 28.8 | 0.904 | 7.89 | −0.25 |
| Pentachlorophenol | 87-86-5 | 266.34 | 20 | 1.33 | 20.1 | 19.04 | NA |
| Isoeugenol | 97-54-1 | 164.21 | 1.35 | 12.2 | 1.70 | NA | −1.24 |

**Supplementary Table 3**. Correlation coefficient comparing the different assay’s potency measure to LLNA on overlapping set of chemicals. Set 1 corresponds to chemicals with data in both GARDskin Dose-Response and hCLAT, set 2 to chemicals with data in both GARDskin Dose-Response and kDPRA, and set 3 to chemicals with data in all three assays.

|  | **Measure** | **Pearson correlation** | **p-value correlation** | **N** |
| --- | --- | --- | --- | --- |
| Overlapping set 1 | GARDskin cDV_0_ | 0.823 | 2.97×10^-4^ | 14 |
|  | hCLAT MIT | 0.799 | 6.07×10^-4^ | 14 |
| Overlapping set 2 | GARDskin cDV_0_ | 0.875 | 1.95×10^-4^ | 12 |
|  | kDPRA log k_max_ | 0.753^*^ | 4.74×10^-3^ | 12 |
| Overlapping set 3 | GARDskin cDV_0_ | 0.874 | 4.35×10^-4^ | 11 |
|  | hCLAT MIT | 0.882 | 3.32×10^-4^ | 11 |
|  | kDPRA log k_max_ | 0.752^*^ | 7.63×10^-3^ | 11 |

* The correlation for kDPRA’s log kmax with LLNA was based on pEC3 values (as described in ^1^), because the assay’s potency measure is in units of s^-1^M^-1^.

hCLAT MIT values were extracted from the Cosmetics Europe database ^2^.

kDPRA log k_max_ values were obtained from ^1^.

1 Natsch, A., Haupt, T., Wareing, B., Landsiedel, R. & Kolle, S. N. Predictivity of the kinetic direct peptide reactivity assay (kDPRA) for sensitizer potency assessment and GHS subclassification. *Altex* **37**, 652-664, doi:<https://doi.org/10.14573/altex.2004292> (2020).

2 Hoffmann, S. *et al.* Non-animal methods to predict skin sensitization (I): the Cosmetics Europe database<sup/>. *Crit Rev Toxicol* **48**, 344-358, doi:<https://doi.org/10.1080/10408444.2018.1429385> (2018).
